# Experimental Study on the Fire Properties of Nitrocellulose with Different Structures

**DOI:** 10.3390/ma10030316

**Published:** 2017-03-20

**Authors:** Ruichao Wei, Yaping He, Jiahao Liu, Yu He, Wenzhong Mi, Richard Yuen, Jian Wang

**Affiliations:** 1 State Key Laboratory of Fire Science, University of Science and Technology of China, Hefei 230026, China; rcwei@mail.ustc.edu.cn (R.W.); jhliu12@mail.ustc.edu.cn (J.L.); hy2015@mail.ustc.edu.cn (Y.H.); 2 Department of Civil and Architectural Engineering, City University of Hong Kong, Hong Kong 999077, China; bckkyuen@cityu.edu.hk; 3 School of Computing, Engineering and Mathematics, University of Western Sydney, Sydney 1797, Australia; Y.HE@uws.edu.au; 4 Fire Department of Ministry of Public Security, Beijing 100054, China; wrcustc@gmail.com

**Keywords:** nitrocellulose, cone calorimeter, combustion characteristic, heat release rate intensity, hazard assessment

## Abstract

In order to ensure the safety of inflammable and explosive chemical substance such as nitrocellulose (NC) mixtures in the process of handing, storage, and usage, it is necessary to obtain the fire properties of NC with different exterior structures. In present study, fire properties of two commonly used nitrocelluloses with soft fiber structure and white chip structure were investigated by scanning electron microscope (SEM) and the ISO 5660 cone calorimeter. Experimental findings revealed that the most important fire properties such as ignition time, mass loss rate and ash content exhibited significant differences between the two structures of NC. Compared with the soft fiber NC, chip NC possesses a lower fire hazard, and its heat release rate intensity (HRRI) is mainly affected by the sample mass. In addition, oxygen consumption (OC) calorimetry method was compared with thermal chemistry (TC) method based on stoichiometry for HRRI calculation. HRRI results of NC with two structures obtained by these two methods showed a good consistency.

## 1. Introduction

Nitrocellulose (NC), which tends to be spontaneously ignited and deflagrable, has been widely used in military and civilian industry to produce explosives, lacquers, films and celluloid products [1]. This material self-ignites easily when directly exposed to a hot and/or humid environment. One typical disastrous accident involving NC occurred in Tianjin Port in China on 12 August 2015, in which two severe explosions were triggered, causing 165 deaths, 798 injuries, and 8 missing. The direct economic loss was CNY 6.866 billion [2]. Thus, the study of fire behavior of NC is necessary and fire hazards of such materials must be evaluated.

Previous work on NC mainly focused on heat of combustion [3], spontaneous ignition [4,5], morphological behavior [6,7,8,9,10] and thermal decomposition [1,11,12,13,14,15,16,17,18,19,20]. Sovizi et al. [20] investigated the thermal stability of micron- and nano-sized NC by simultaneous thermogravimetry-differential thermal analysis (TG/DTA) techniques. It was concluded that particle size of NC could affect its thermal stability and its decomposition temperature decreases with decreasing particle size. Although numerous studies of NC exist, there has been relatively little work conducted to examine the effect of exterior structures on fire characteristics of NC, such as ignition time and heat release rate intensity (HRRI), or the heat release rate (HRR) per unit of exposed surface area under given external radiant heat flux. The ISO 5660 cone calorimeter test is a kind of conventional method which has been widely used to measure the combustibility of materials. However, no relevant literature was found to employ this apparatus to investigate the ignition and burning characteristics of NC.

**Figure 1:**
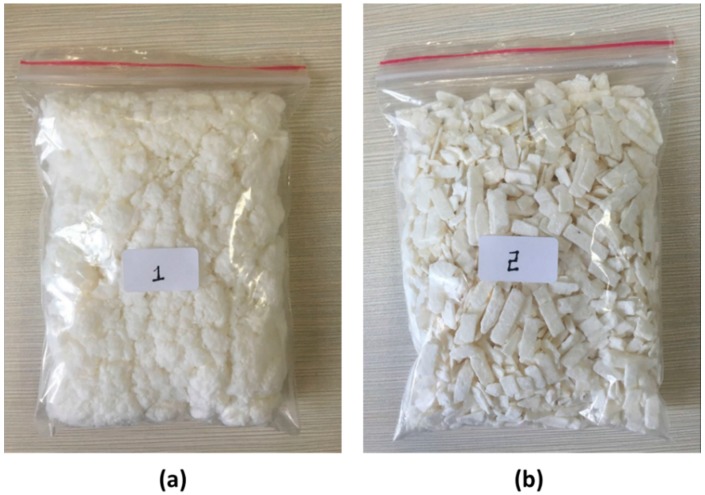
Comparison of the macroscopic product structures. (**a**) White soft fiber nitrocellulose (NC-F); (**b**) white chip nitrocellulose (NC-C).

Pure nitrocellulose (C_12_H_16_N_4_O_18_) appears in white fibers on its own. However, it is usually mixed with other substances to take different forms or structures to satisfy the practical needs of different fields of industry. White soft fiber NC (NC-F) and white chip NC (NC-C) are two of the most common forms. The NC-F form appears to be in lumps of loosely packed fibers much like cotton balls (see Figure 1a). Given its high potential of spontaneous combustion, NC-F is often mixed with some humectants such as water or alcohol to improve its stability in the process of production, storage and transportation. The NC-C on the other hand is a mixture of NC fiber and some plasticizer compressed together to form chips (Figure 1b) which do not deform easily when point pressure is applied. Compared with NC-C, NC-F exhibits characteristics of high gloss and less dust, and it is easy to store. More detailed properties of the two forms of NC are given in Section 2.1.

It is well-known that the physical form of solid fuels affects their ignition and burning characteristics. For example, wood of the same species in sawdust, cribs and solid timber forms will burn differently [21,22,23]. Given a right environment, sawdust can be explosive whereas at the other extreme, a piece of bulky solid timber is difficult to ignite and once ignited, the fire is difficult to sustain without an additional external heat source [23]. On the same token, it can be hypothesized that the ignition and burning behavior of NC-F and NC-C will be quite different. In order to identify and understand the possible differences, and in order to enhance the strategies and procedures for the manufacturing, transport and storage of the different forms of NC, a comprehensive investigation of their fire properties are urgently needed.

The aim of the present study is to meet this need by experimental means. Two experimental apparatus, namely the scanning electron microscope (SEM) and the cone calorimeter, were employed to reveal the differences in the microscopic structures and in the thermal and fire properties of the two NC samples under various conditions. The fire properties include the ignition time and the burning intensity. The results of NC-F are compared with those of NC-C.

## 2. Experimental Description

### 2.1. Samples

NC-F and NC-C samples used in this work are produced by Sichuan Nitrocell Co., Ltd. (Luzhou, China). The physical parameters of the two samples obtained from the product brochure are listed in Table 1. The macroscopic product structures of the two samples are shown in Figure 1. NC-F has a soft surface and the fibers integrated with each other to form conglomerations, while NC-C presents a fixed strip structure. In addition to the exterior structure, the major difference for the two samples is that NC-F contains 29 wt % humectant isopropanol (C_3_H_8_O) while NC-C contains 19.5 wt % plasticizer dibutyl phthalate (C_16_H_22_O_4_).

### 2.2. Apparatus

#### 2.2.1. SEM

A scanning electron microscope (SEM) is used to distinguish the difference in physical microcosmic structures of the two NC samples. Scanning electron micrographs were recorded employing the Philips XL30 ESEM-TMP SEM made in Netherlands Philips. The scope of magnification is from 6 to 1,200,000 times, and the accelerating voltage of the SEM is 20 kV.

#### 2.2.2. ISO 5660

The ISO 5660 cone calorimeter was exploited to test the ignition time, HRRI, and mass variation of the NC with different structures. The oxygen consumption calorimetry technique [24] was employed in this work. From ISO 5660-1 [25], the HRR is calculated as follows:

(1) $$HRR=1.10EX_{O_{2}}^{0}\left[\phi /\left[\left(1-\phi \right)+1.105\phi \right]\right]{\dot{m}}_{e}$$

(2) $$\phi =\left[X_{O_{2}}^{0}\left(1-X_{CO_{2}}\right)-X_{O_{2}}\left(1-X_{CO_{2}}^{0}\right)\right]/\left[X_{O_{2}}^{0}\left(1-X_{CO_{2}}-X_{O_{2}}\right)\right]$$

(3) $${\dot{m}}_{e}=C\sqrt{\Delta p/T_{e}}$$

where E is the heat of combustion per unit mass of oxygen consumed, ϕ is the oxygen consumption factor defined in Equation (2), ${\dot{m}}_{e}$ is the mass-flow rate in the exhaust duct defined in Equation (3), C is the orifice plate calibration constant, Δp is the orifice meter pressure differential, $T_{e}$ is the absolute temperature of the gas at the orifice meter, $X_{A}^{0}$ is the initial concentration of species *A*, and $X_{A}$ is the output data of gas analyzers. The environmental H_2_O concentration was neglected because the exhaust gas passes through a calcium chloride filter. CO concentration was also neglected because of its relative low concentration during flaming combustion.

The heat release rate intensity (HRRI, kW·m^−2^) is obtained from

(4) $$HRRI=HRR/A_{c}$$

where A_c_ is the top opening area of the sample crucible.

### 2.3. Experimental Setup of the Cone Calorimeter

**Figure 2:**
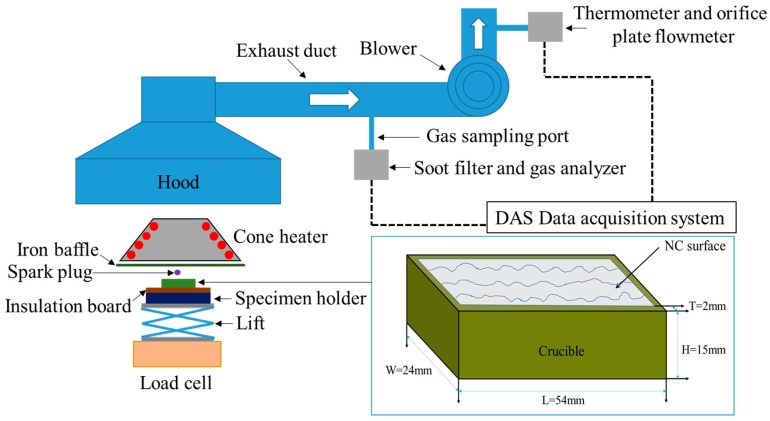
Schematic of the experimental setup.

The experimental setup of the cone calorimeter in the current study complied with ISO 5660 [25]. Figure 2 presents a schematic of the experimental setup. An iron baffle is horizontally arranged under the cone heater to prevent the samples from being affected by the radiant heat flux before the commencement of the tests. An insulation board with dimensions of 100 mm × 100 mm × 10 mm (length × width × height) was positioned on the conventional specimen holder to shield the scale from the elevated temperature. The NC samples were put into a cuboid ceramic crucible which is shown in amplified part of Figure 2 and the crucible was placed horizontally on the insulation board. The crucible is 15 mm in height, 2 mm in thickness, and has an open rectangular area of 1000 mm^2^. For each test, initial sample surface area keeps consistent. Standard area mentioned in ISO 5660 [25] is 94 × 94 = 8836 mm^2^. Previous research has shown that a reduction in the surface area of cone calorimeter test specimens does not significantly affect their fire behavior [26]. A lift was positioned under the specimen holder for adjusting the distance between the upper surface of the sample and the base of the cone heater. A spark plug was positioned above the sample surface for piloted ignition. An electronic scale (Shimadzu UW 6200H) with weighing capacity of 6200 g and accuracy of 0.01 g was placed at the bottom to monitor the mass loss of the sample. The calibration of each part of the cone calorimeter is carried out according to ISO 5660 [25].

### 2.4. Experimental Conditions and Procedure

**Figure 3:**
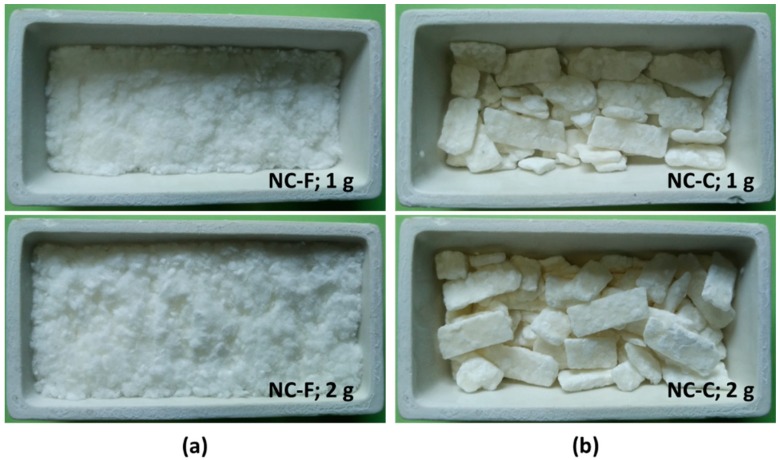
Photos of crucibles loaded with 1 g and 2 g samples. (**a**) NC-F; (**b**) NC-C.

To study the ignition and burning behavior of the two forms of nitrocellulose or nitrocellulose mixtures, the experimental conditions in current study are listed in Table 2. Before each test, the NC-F or NC-C sample was spread and forced compressed in the crucible to deduce the air gap and to achieve similar bulk density, even distribution and level surface as shown in Figure 3. The initial upper surface of the samples at the beginning of each test was at the standard distance of 25 mm below the base of the cone heater. For different mass samples, the height of the lift is adjusted to ensure a constant distance between the initial upper surface of the samples and the base of the cone heater. NC has an explosive hazard [1], thus only small amount of sample was used in each test as a safety precaution. To study the influence of external heat exposure, the heat flux level of the cone heater was set at 0, 5, 10, 15 and 20 kW/m^2^ in different tests. In each test, the cone heater was preheated to the desired irradiance level before the sample was placed underneath. In the case of zero external heat flux, the spark plug was replaced with a propane cigarette ignitor. The flame of the ignitor acted as a heat source as well as the ignitor. The ignitor was removed from the sample after the ignition.

When the iron baffle was removed and the spark plug was switched on, the program in the computer was activated to record the experimental data. Once the visible flame was observed, the sample was considered ignited. The ignition spark was switched off and removed from the sample surface once the sample was ignited. If the sample was not ignited within 600 s, the experiment was forcibly ended. Each test was repeated three times, and the final results presented in this paper are the average values of the repeated tests.

## 3. Results and Discussion

### 3.1. Microcosmic Structures

**Figure 4:**
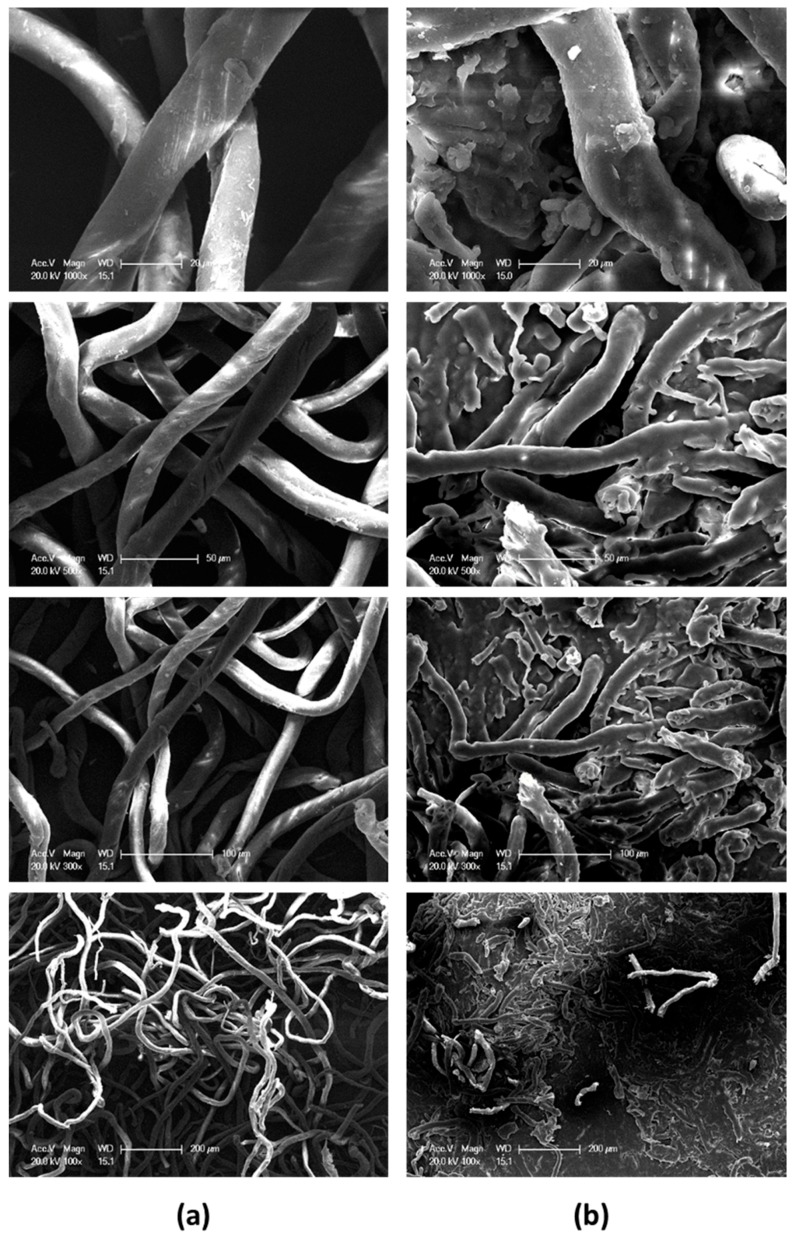
Comparison of the microcosmic structures. (**a**) NC-F; (**b**) NC-C.

The microcosmic structures of four different magnifications, examined by scanning electron microscope (SEM), are exhibited in Figure 4. At the high magnification (20 μm scale) NC-F appears smooth surface except some minor flaws and NC-C presents its self in a molten and adhesion state. The diameter of the fibers in both structures is in the proximity of 20 μm. At the medium magnifications (50 μm and 100 μm scales), the twining fibrous micro structures are easily observed for NC-F, while the fibers in NC-C appear to be segmented. The space between the fibers in NC-F is observably larger than in NC-C. At the low magnification (200 μm scale), the contrast between the two structures is even greater. The agglomeration of NC fiber in NC-C is much denser than in NC-F. It is noted that the SEM images in Figure 4 are very similar to that of micron-sized NC fibers examined by Sovizi et al. [20].

### 3.2. Combustion Characteristics

#### 3.2.1. Ignition Time

Ignition time is defined as the time interval between the onset of exposure to external heat flux and the appearance of continuous flame on the sample surface. The latter is associated with the significant rises in mass loss rate intensity (MLRI) and/or heat release rate intensity (HRRI). The measured ignition time of the two tested samples as functions of irradiance are listed in Table 3.

Table 3 shows that ignition times of the two NC forms can be dependent on the initial sample mass. For samples with less mass, the exposed edge of crucible above the upper surface of NC mixtures is higher, thus the crucible will block more heat transfer and increase the ignition time. Generally, the greater the initial mass is, the shorter the ignition time. There is one exception where it took longer for the NC-F sample of 2 g to ignite than for its companion of 1 g under the irradiance of 15 kW/m^2^. Compared with NC-F, the differences in the ignition times between the 1-g and 2-g NC-C samples are much smaller. Besides, NC-F exhibits higher uncertainty over the repeated tests for the case with lower external radiation (kW/m^2^). It is also seen from Table 3 that the ignition time of NC-C is always greater than that of NC-F under the identical initial mass and external heat flux conditions. The reason might be that the volatilization of isopropyl alcohol affects the ignition time and low flash point of isopropyl alcohol (12 °C) contributes to the combustion of NC-F. The most important conclusion drawn from Table 3 is that the ignition time decreases with the increasing external radiant heat flux for the two samples, regardless of the mass of the sample.

Ignition time of a combustible material is determined by the thermal and fire properties of the material and the external thermal environment to which the material is exposed. One of the determining fire properties is the ignition temperature, or the ignition point or fire point [27]. The critical heat flux (CHF) is another important parameter to determine the ignition characteristic of combustibles. It is the minimum external heat flux applied to the combustible material surface in order for ignition to occur.

Based on the concept of heat balance, the portion of the critical heat flux absorbed by the material is quantitatively equal to the heat loss from the combustible material surface to the surrounding environment at the ignition point expressed as follows [28,29]:

(5) $$CHF=\sigma T_{ign}^{4}+\left(h_{c}/\varepsilon \right)\left(T_{ign}-T_{0}\right)$$

where ε, *h_c_*, *T_ign_*, *T*_0_ and σ is the emissivity of the material, the convective heat transfer coefficient, the ignition temperature, the ambient temperature and the Stefan–Boltzmann constant, respectively.

Previous research [30,31,32] has also deduced a classical formula to describe the relationship between ignition time and external heat flux. That is, for the thermally-thick materials, $1/\sqrt{t_{ign}}$ is proportional to the external radiant heat flux ${\dot{q}}_{ext}^{\prime \prime }$, i.e.,

(6) $$1/\sqrt{t_{ign}}=\left(2{\dot{q}}_{ext}^{\prime \prime }\right)/\left[\sqrt{\pi k\rho c}(T_{ign}-T_{0})\right]$$

where $t_{ign}$, k, ρ and c is the ignition time, the thermal conductivity, the density, and the specific heat, respectively. Combining Equations (5) and (6), the resulting equation can be written in a linear form:

(7) $$y=1/\sqrt{t_{ign}}=a\left({\dot{q}}_{ext}^{\prime \prime }-CHF\right)=a{\dot{q}}_{ext}^{\prime \prime }-b$$

where,

(8) $$a=\varepsilon /\left[\sqrt{\left(\pi /4\right)k\rho c}\left(T_{ign}-T_{0}\right)\right]$$

and,

(9) b=aCHF

or,

(10) CHF=b/a

Equation (9) allows the estimate of *CHF* from the line fitting parameters *a* and *b*.

**Figure 5:**
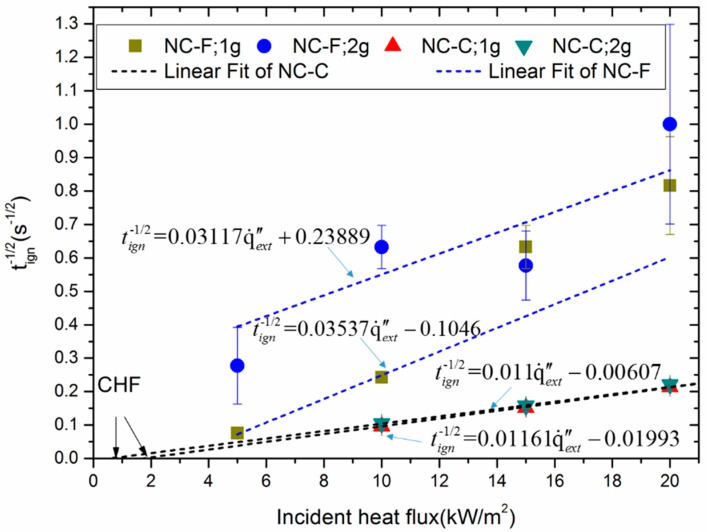
The plots of $t_{ign}^{-1/2}$ versus incident heat flux for different samples. CHF: critical heat flux.

By analyzing the experimental data, the correlation between $t_{ign}^{-1/2}$ and external radiant heat flux can be derived according to the linear fit of Equation (6) to the data in Figure 5. The results are summarized in Table 4.

Figure 5 shows that the linear fitting between $t_{ign}^{-1/2}$ and incident heat flux is unsuccessful for NC-F. The results of NC-C fit well with Equation (6) and the corresponding CHF for 1 g NC-C and 2 g NC-C are 0.55 kW/m^2^ and 1.72 kW/m^2^, respectively. The difference of the CHF is attributed to the variation of the convective coefficient as shown in Equation (5). Besides, the ignition times of 1 and 2 g of NC-C, with external radiation of 5 kW/m^2^ predicted from the fitting line are 688 s and 418 s, respectively. However, 2-g NC-C samples under the actual measurement were not ignited within 600 s. This may be attributed to air convection produced by the open cone calorimeter affecting the heat accumulation of the samples.

#### 3.2.2. Mass Loss and Ash Content

**Figure 6:**
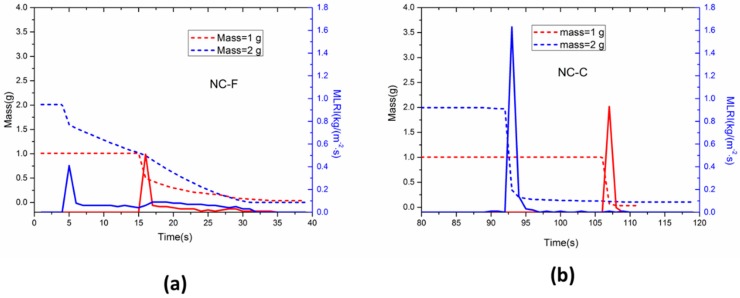
Mass loss rate intensity profiles of (**a**) NC-F and (**b**) NC-C under 10 kW/m^2^.

The typical mass loss rate intensity (MLRI) curves of two NC samples at 10 kW/m^2^ are shown in Figure 6. A high peak of MLR was recorded immediately after spark ignition. The flaming combustion consumed 20–50% of the initial mass for NC-F and 80–90% for NC-C. The peak mass loss rate intensity (PMLRI) of NC-C is much greater than that of NC-F. The burning period of NC-C was very short, in a matter of less than 3 s. The burning period of NC-F seemed to last much longer, for about 20 s or longer.

Ash content is defined as the percentage residue after combustion. Ash content and its standard deviation under different experimental conditions are listed in Table 5. The following information can be extracted from Table 5. Firstly, for NC-F with two masses, ash contents show a relatively stable value for different radiant heat fluxes. However, it is worthwhile to note that more ash content was observed for the 1-g sample. Secondly, for NC-C with two masses, complete combustion can be observed, and ash contents exhibit a decreasing trend with increasing radiant heat flux. Meanwhile, for 2-g NC-C, ash content is higher than that of 1 g, which is contrary to NC-F.

#### 3.2.3. Heat Release Rate

**Figure 7:**
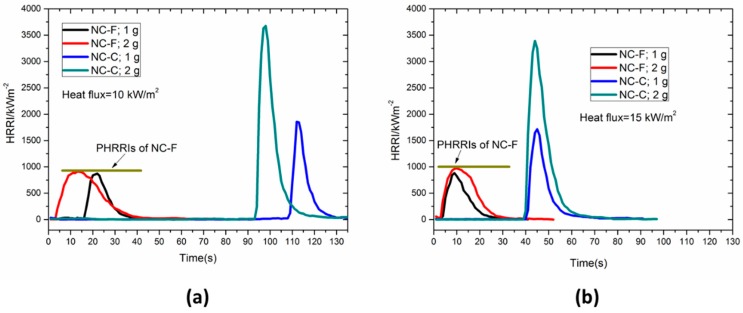
Transient evolution of heat release rate (HRR) for samples with different masses. (**a**) Heat flux: 10 kW/m^2^; (**b**) heat flux: 15 kW/m^2^. HRRI: heat release rate intensity; PHRRI: peak heat release rate intensity.

The heat release rate intensity (HRRI) histories of NC samples with initial masses of 1 and 2 g are plotted in Figure 7. It is found that regardless of initial mass, NC-F tends to reaches the same peak heat release rate intensity (PHRRI) under 10 kW/m^2^ and 15 kW/m^2^ and the PHRRI is about 1000 kW/m^2^. Compared with NC-F, NC-C does not exhibit the same PHRRI under different external radiant heat fluxes. Its PHRRI is significantly greater than that of NC-F under the same sample mass and heat flux conditions. In addition, the PHRRI of NC-C exhibits a strong mass dependence. The PHRR of the 2-g sample is roughly 2 times that of the 1-g sample. The reason might be that, compared with NC-F, the flame of NC-C produces higher radiation than external radiation and the flame radiation increases with the sample mass.

**Figure 8:**
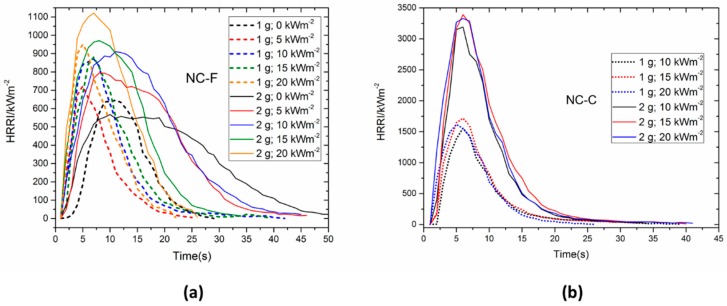
Comparisons of HRRI profile under different heat fluxes. (**a**) NC-F; (**b**) NC-C.

The trends of HRRI for NC-F and NC-C under different external heat fluxes are plotted in Figure 8, where the ignition time is omitted and all the curves uniformly commence from the ignition time. The NC-F samples were ignited by a propane fire when the external heat flux is absent. Only one peak values can be observed for all the tests. The PHRRI of NC-C is far larger than that of NC-F under any given irradiance. The HRRIs of NC-C increased very fast to approach the peak value shortly after the piloted ignition, then decreased quickly. However, the HRRIs of NC-F decreased gradually after the peak value, which indicates that the wetting agent isopropanol can effectively suppress the rapid combustion of the NC.

It can be seen from Figure 8a that the PHRRI of NC-F increases with increasing external radiant heat flux, and the increasing rate also goes up with the increasing radiant heat flux. With larger radiant heat flux, the initial growth rate of HRR, or *d*(HRR)/*dt*, and the PHRRI become higher, and the combustion duration becomes shorter. In Figure 8b, similar curves of HRRI is observed for a fixed mass under three different radiant heat fluxes. The HRRIs of NC-C have a weak relationship with external heat flux, but are affected significantly by the sample mass.

**Figure 9:**
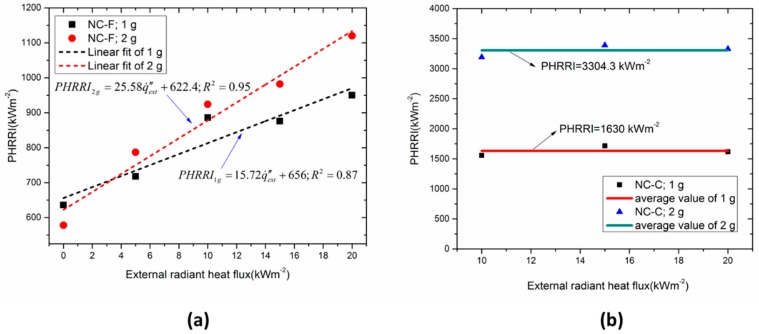
PHRRI as a function of external heat flux. (**a**) NC-F; (**b**) NC-C.

Figure 9 presents the trends of PHRRI with the increasing external radiant heat flux. Generally, the magnitude of PHRR of NC-C is larger than that of NC-F. The PHHRI of NC-F increases as a function of external radiant heat flux. By linear fitting, the relationship between PHRR and external radiation for NC-F can be expressed as:

(11) $$PHRRI_{1g}=15.72{\dot{q}}_{ext}^{\prime \prime }+656;\;R^{2}=0.87$$

(12) $$PHRRI_{2g}=25.58{\dot{q}}_{ext}^{\prime \prime }+622.4;\;R^{2}=0.95$$

For NC-C, the PHRRI appears to be independent of the irradiance but dependent on the initial sample mass (Figure 9b). This latter dependence can be expressed as:

(13) $$PHRRI\approx 1630m_{0}$$

where *m*_0_ is the initial sample mass (g).

### 3.3. Hazard Assessment

In order to investigate the flashover propensity of the materials, Petrella [33] proposed a parameter *x*, which is defined as:

(14) $$x=\frac{PHRR}{t_{ign}}$$

Standardization of parameter *x* for risk classification of materials is listed in Table 6 [30,34]. The experimental results of *x* for NC-F and NC-C are listed in Table 7 and the corresponding fire hazards for different configurations are evaluated based on Table 6. The average *x* is above 10, indicating that NC-F and NC-C are seriously hazardous. Based on the same mass for different NCs, NC-F presents a higher fire risk. Meanwhile, the fire risk of both NC-F and NC-C increase with the increasing external radiant heat flux. The fire risk of NC-F is strongly influenced by external radiant heat flux and its risk level markedly increases when the external heat flux is around 10 kW∙m^−2^. However, for NC-C, an increased fire risk occurs when the external heat flux is 20 kW∙m^−2^. In addition, for different masses of the same NC, fire risk of 2-g samples are much higher than for 1-g samples, indicating that the fire risk increases with the increasing sample mass.

### 3.4. Verification on the Validity of Tested HRR Data

In order to verify the validity of the HRR data obtained by cone calorimeter, thermal chemistry (TC) method is employed in this article. Based on Hess’s law and Lavoisier law, heat release in a chemical reaction can be calculated from the difference of the enthalpy of the formation $\Delta H_{f}^{o}$ per unit mole between the products and reactions [27], i.e.,

(15) $$q=\sum_{products=i}\left\lfloor {\left(\Delta H_{f}^{o}\right)}_{i}\cdot {\dot{n}}_{i}\right\rfloor -\sum_{reactants=j}\left\lfloor {\left(\Delta H_{f}^{o}\right)}_{j}\cdot {\dot{n}}_{j}\right\rfloor$$

where *q* is the total heat released in a combustion process, and *n* is the relative mole amount of chemicals.

For a typical fuel which contains only C, H, O and N atoms, the chemical reaction can be represented as:

(16) $$aC_{w}H_{x}O_{y}N_{z}+1O_{2}\to A{\mathrm{CO}}_{2}+b\mathrm{CO}+cH_{2}O+dN_{2}$$

where coefficient *A* represents the amount of CO_2_ produced when every unit mole of oxygen is consumed, and it equals the ratio of increment of CO_2_ to decrement of O_2_. Coefficients *a*, *b*, *c*, and *d* represent the relative amount of fuel, CO, H_2_O, and N_2_, respectively. Based on the conservation of C, H, O, and N atoms before and after the reaction, *a*, *b*, *c* and *d* can be calculated as Equation (17).

(17) $$\left\{ \begin{array}{l} ay+2=2A+b+c\\ a=\left(A-2\right)/\left[y-w-\left(x/2\right)\right]\\ b=w(A-2)/\left[y-w-\left(x/2\right)\right]-A\\ c=x(A-2)/\left[2\left(y-w-\left(x/2\right)\right)\right]\\ d=z(A-2)/\left[2\left(y-w-\left(x/2\right)\right)\right] \end{array}\right.$$

Combined with the Equations (15) and (16), the heat released by consuming unit mole of oxygen in a chemical reaction can be expressed as:

(18) $$q=A\cdot \Delta H_{f,{\mathrm{CO}}_{2}}+b\cdot \Delta H_{f,\mathrm{CO}}+c\cdot \Delta H_{f,H_{2}O}-a\cdot \Delta H_{f,C_{w}H_{x}O_{y}N_{z}}$$

If the mass production rate intensity of carbon dioxide ${\dot{m}}_{{\mathrm{CO}}_{2}}^{\prime \prime }$ is known, the heat release rate intensity can be presented as:

(19) $$HRRI=\frac{q}{A}\cdot \frac{{\dot{m}}_{{\mathrm{CO}}_{2}}^{\prime \prime }}{44}$$

where ${\dot{m}}_{{\mathrm{CO}}_{2}}$ is the mass flow rate of carbon dioxide in the exhaust gases, and this data can be obtained from the output of the cone calorimeter test.

In order to get the values of *w*, *x*, *y* and *z*, assumptions were made that the mixtures can be treated as a single substance based on the proportion of the C, H, O, and N atoms. Thus, the equivalent formula for the NC-F was derived by the combination of C_3_H_8_O (isopropyl alcohol and relative molar mass M_1_ is 60 g·mol^−1^) and C_12_H_16_N_4_O_18_ (pure NC and relative molar mass M_2_ is 504 g·mol^−1^). The mass ratio of the two is 29:71, thus the molar mass ratio can be calculated as 14,616/420 and we get 3.43/1. Then, the number *w* can be deduced as *w* ≈ (3.43 × 3 + 1 × 12)/(3.43 + 1) = 5.03. In the same way, *x*, *y* and *z* are calculated to be 9.81, 4.84 and 0.9, respectively. Therefore, the equivalent formula of the NC-F is C_5.03_H_9.81_O_4.84_N_0.9_. Similarly, the equivalent formula for the NC-C is C_2.82_H_4_O_3.06_N_0.85_. The *A* and *q* values for NC-F and NC-C are given in Table 8.

**Figure 10:**
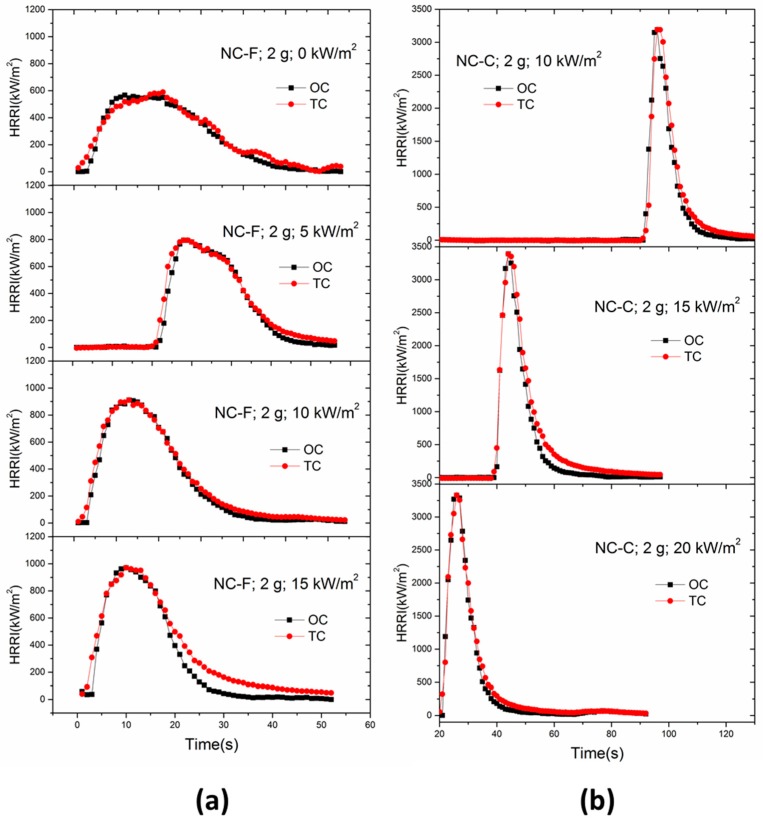
HRR profiles calculated by OC and TC methods. (**a**) NC-F-2 g; (**b**) NC-C-2 g.

Assuming that the combustion in the cone calorimeter experiment was complete or stoichiometric, and the carbon dioxide concentration in the ambient air is negligible, then HRRI can be calculated from the parameters given in Table 8 and the measured carbon dioxide mass flow rate. This method is denoted as the thermal chemistry method (TC). The results are compared in Figure 10 with the cone calorimeter output HRRI, which is based on Equations (1)–(3) and is denoted as the oxygen consumption method (OC). The HRRI curves obtained by two methods are in good agreement except that, on the whole, *Q_TC_* is slightly higher than *Q_OC_*. This is because that the TC method takes into account the participation in the combustion by the oxygen in NC mixtures. On the other hand, the parameter *E* in Equation (1) is an empirical constant which does not reflect the variations of oxygen content in the reactants. It is likely that the E value used in the cone calorimetry underestimated the contribution from the oxygen in the NC mixture samples to the HRRI. Besides, because of the good agreement between the estimated heat release rate intensities based on the two methods, the cone calorimeter is considered to be an appropriate apparatus for measuring the heat release rate of the two NC mixtures.

## 4. Conclusions

The fire characteristics of NC samples with different exterior structures were investigated employing SEM and the cone calorimeter in this study. Specimens with different masses were tested under various external radiant heat fluxes. The ignition time, HRRI, MLRI, ash content and hazard assessment were conducted. The following conclusion can be made:

1. The ignition time of NC-F is shorter than that of NC-C under the range of the external radiant heat flux tested (10–20 kW·m^−2^). The ignition time of NC-C agrees well with the existing ignition model.
2. Although the ignition times of NC-F were quite sporadic and did not conform to the existing model, its ignition time was found to be shorter than that of NC-C under the same radiant heat exposure.
3. The heat release rate intensity of both forms of nitrocellulose was found to be dependent on the sample mass. The HRRI of NC-F is also affected by the external radiant heat flux.
4. Compared with NC-F, NC-C has a higher PMLRI and less ash content.
5. Based on the *x*-factor evaluation, NC-F sample was found to possess a higher fire hazard than NC-C.
6. A TC method based on stoichiometry was employed to study the validity of the HRR obtained from the cone calorimeter. The HRRI results of the two methods were found to be in good consistency. Thus, oxygen consumption calorimetry is considered to be an appropriate technique to determine the HRRI in fires in relation to NC-F and NC-C.

## Figures and Tables

**Table 1:** Physical parameters of samples.

Material	NC-F	NC-C
Apparent density (kg/m^3^)	250	600
Nitrogen content (%)	12.00	11.96
Plasticizer and content	-	Dibutyl phthalate (DBP) 19.5 wt %
Humectant and content	Isopropanol 29%	-
Acidity (as H_2_SO_4_) (%)	0.04	0.03
Ignition point (°C)	182	174
Viscosity (s)	3.3	5.2
80 °C Thermal-resistance test (min)	15	>15

**Table 2:** The experimental configurations.

Sample	External Heat Flux (kW/m^2^)	Initial Mass (g)	Ambient Temperature (°C)	Ambient Relative Humidity (%)
NC-F	0, 5, 10, 15, 20	1 ± 0.02	15 ± 2	45 ± 5
		2 ± 0.04		
NC-C	0, 5, 10, 15, 20	1 ± 0.02		
		2 ± 0.04		

**Table 3:** Ignition times corresponding to two samples.

Heat Irradiance (kW/m^2^)	Ignition Time (s)			
	NC-F		NC-C	
	1 g	2 g	1 g	2 g
0	I	I	N_1_	N_1_
5	176.5 ± 30.5	13 ± 8	N_2_	N_2_
10	17 ± 1	2.5 ± 0.5	108 ± 1	92 ± 1
15	2.5 ± 0.5	3 ± 1	43.5 ± 3.5	40.5 ± 0.5
20	1.5 ± 0.5	1 ± 0.5	22 ± 1	20.5 ± 0.5

I: The samples were instantly ignited by the propane cigarette ignitor; N_1_: The samples cannot be ignited by the propane cigarette ignitor; N_2_: The samples cannot be ignited by heat irradiance within 600 s.

**Table 4:** Liner regression results of the data in Figure 5.

Sample	*a* (m^2^·kW^−1^·s^−1/2^)	*b* (s^−1/2^)	*R*^2^
NC-F, 1 g	0.03537	−0.1046	0.9459
NC-F, 2 g	0.03117	0.23889	0.30557
NC-C; 1 g	0.01161	−0.01993	0.99933
NC-C; 2 g	0.011	−0.00607	0.99423

**Table 5:** Recorded ash content (%) of individual tests and the basic statistics.

Test No.	${\dot{q}}_{ext}^{\prime \prime }$ (kW·m^−2^)	NC-F		NC-C	
		1 g	2 g	1 g	2 g
1	5	8.18	2.39	-	-
2	5	5.09	2.20	-	-
3	5	3.26	4.08	-	-
4	10	4.95	1.59	1.88	3.24
5	10	5.85	2.34	4.55	3.76
6	10	6.72	1.00	3.29	1.10
7	15	4.67	2.50	0.90	1.75
8	15	4.00	2.10	0.30	1.89
9	15	3.48	2.05	1.00	1.34
10	20	5.33	0.60	0.20	0.50
11	20	5.19	1.00	0.20	0.15
12	20	2.39	1.55	0.30	1.60
Total number of tests of the same sample		12	12	9	9
Mean		4.93	1.91	1.40	1.70
Standard deviation		1.50	0.90	1.47	1.11

**Table 6:** Parameter *x* for predicting fire risk [30,34].

Values	Risk Classification
0.1–1.0	Low risk
1.0–10	Intermediate risk
10–100	High risk
100–1000	Very high risk

**Table 7:** Risk classification of NC samples under different experimental conditions.

*q*″	1 g				2 g			
	NC-F		NC-C		NC-F		NC-C	
	*x*	Classification	*x*	Classification	*x*	Classification	*x*	Classification
5	4.07	Low	-	-	60.54	High	-	-
10	52.12	High	14.42	High	369.60	Very high	34.69	High
15	584	Very high	39.45	High	327.33	Very high	83.73	High
20	601.36	Very high	73.5	High	971.62	Very high	164.44	Very high

**Table 8:** The *A* and *q* values for NC-F and NC-C.

Material	*A*	*a*	*b*	*c*	*d*	*q*
NC-F	0.84	0.22	0.31	1.12	0.10	477.40
NC-C	0.63	0.78	1.57	1.56	0.33	2248
